# A Novel Aberrant HbF Peak with Electrophoretic Shift in A1c of a Patient with Chronic Lymphocytic Leukemia (CLL) Was Reversible to Give Interpretable Results

**DOI:** 10.3390/biomedicines14010171

**Published:** 2026-01-13

**Authors:** Mark E. Obrenovich, Elizabeth A. Schroer, Yi Li, Ronald Quam, Angel Munoz, Shagufta Khan

**Affiliations:** 1 Cincinnati Veterans Affairs Medical Center, Cincinnati, OH 45220, USA; 2 The Gilgamesh Foundation, Cleveland, OH 44116, USA; 3 Department of Health, Nursing and Nutrition, University of the District of Columbia, Washington, DC 20008, USA; yi.li@udc.edu

**Keywords:** capillary electrophoresis, hemoglobin A1c, Chronic Lymphocytic Leukemia

## Abstract

**Background**: A strikingly unusual pattern with a possible up-field shift in Hemoglobin A1c (HbA1c) and A0 (HbA0) peaks and an unexplained hemoglobin F (HbF) peak with capillary electrophoretic shift in the HbA1c chromatograms of a leukemia patient were found while performing a HbA1c screen. **Methods**: A review of the patient’s history with an exhaustive search of the literature ruled out medications as interfering factors or contributing to the abnormal findings. Other than hyperleukocytosis, the patient did not have the aberrant HbF peak noted previously in the electrophoresis or contributing factors. We hypothesized that the irregular chromatographic pattern and wrong location of the HbA1c peak, hereafter referred to as the downfield shift in the electrophoretic species, was due to various glycation or fructosamine adducts and derivatives within the HbA1c and A0 protein. **Results**: A literature search offered little guidance. However, the instrument troubleshooting measures suggested a hemoglobin variant or exogenous transfusion as a putative source for the HbF peak, while the downfield shift in the chromatogram remained unexplained.

## 1. Introduction

A highly complex laboratory of our hospital medical facility serves a mostly male and aged veteran population. Here, a 65-year-old Caucasian male with past medical history of adult Chronic Lymphocytic Leukemia (CLL), Type 2 Diabetes Mellitus (T2DM), hypertension, chronic kidney disease, post-traumatic stress disorder, and treated sleep apnea, presented to the hematology clinic to establish care. He was initially diagnosed with CCL nine years prior to the visit. He had received ibrutinib in the past because of worsening anemia and thrombocytopenia with a markedly elevated white blood cell (WBC) count (>200 K). The initial exam resulted in elevated lactate dehydrogenase, mildly elevated uric acid, and elevated potassium cation (K+) (>10 mmol/L). Hematology–oncology was consulted.

Patient history showed hepatomegaly, splenomegaly, and stable macrocytic anemia, as well as lymphadenopathy, on CT scans at the time of the initial exam. A recent bone marrow biopsy revealed extensively affected bone marrow by CLL with poor prognosis and a clonal B cell expressing an unmutated immunoglobulin heavy chain (IGHV) gene, and mutated TP53 and NOTCH1 genes, along with 13q deletion locus by fluorescence in situ hybridization with otherwise normal cytogenetics. Treatment with ibrutinib was begun six years ago, with one interruption due to personal reasons, and was later resumed. Due to disease progression, he was switched to acalabrutinib from ibrutinib. In the last year, the patient was instructed to visit the emergency department after CBC showed anemia (Hgb < 7 g/dL), and was transfused two units of packed red blood.

The patient was assessed for glucose control in the six months prior to HbA1c determination. He was prescribed prochlorperazine 10 mg per dose orally four times per day as needed and loperamide 2 mg 12 times per day as needed. The patient had received 18 units of type O antigen negative blood and red blood cell components. Further, when the patient was assessed for glycemic control, he did have hyperkalemia, hyperleukocytosis, or T2DM. Hyperkalemia, or pseudo-hyperkalemia, is a common phenomenon in CLL, where high WBC counts can lead to falsely elevated K+ concentrations. This is likely due to cell fragility in centrifuged heparinized tubes, where K+ from intracellular compartment is released, which can falsely elevate the K+ levels. Hyperkalemia, or pseudo-hyperkalemia, is a common phenomenon in CLL, where high WBC counts can lead to falsely elevated K+ levels. This is likely due to cell fragility in centrifuged heparinized tubes, where K+ from intracellular compartment is released falsely elevating the K+ concentration. Moreover, lavender vacutainer tubes contain increased K+ concentrations, as reported by Lima-Oliveira et al. [1]. Potassium Ethylenediaminetetraacetic Acid (K-EDTA) contamination of 0.9 mg/mL (approximately 0.307 mmol/L) has increased K+ concentration by 0.6 mmol/L [1], and the blood draw order can affect K+ levels as well [2].

The subsequent electrophoresis of the patient’s HbA1c sample showed the appearance of a putative hemoglobin (Hb) F peak and marked unexplained electrophoretic shift in the A0 peak into zone Seven. There was a second tube collected on the same day from the same patient, which gave no HbA1c result due to zone shift up field, likely due to hyperleukocytosis, since the issue was resolved when the blood samples were diluted with saline, as indicated in the results section. The chromatographic pattern appeared otherwise typical. The hyperkalemia is likely explained, but the HbF could be one of multiple variants or a denatured HbS, as shown in Table 1. More than 1000 hemoglobin variants have been identified; however, not much is known about the effects of most variants on Hb A1c measurement [3]. Genetic variants of Hb, such as hemoglobin S (HbS) and C (HbC) traits, can have Hb derivative modifications, including nonenzymatic carbamylation in patients with renal failure, or can become acetylated when patients take large amounts of salicylic acid (aspirin) or are recovering from acute blood loss or hemolytic anemia, which all may impair glycated Hb results [4].

Alternative forms of glycation were also suspected, which led to requested consultation with the pathologists and clinical chemists in our laboratory. Upon evaluation, the putative causes of the finding may be drug interference, other interference, and acquired HbF from transfused units. Glycohemoglobin is produced via a non-enzymatic reaction between the free aldehyde group of glucose or other sugars and the unprotonated form of free amino groups of hemoglobin on the beta chain and elsewhere in the molecule. The names of hemoglobin A0 and hemoglobin A1a, A1b, and A1c refer to the order of their elution from an ion exchange column. Hemoglobin A1 is the net sum of hemoglobin A1a, b, and c, of which hemoglobin A1c is the major portion. These fast-moving electrophoretic species localized to the A1c and A0 peaks are termed fast hemoglobin species or glycohemoglobin. Normally, for a typical chromatographic pattern, the fitted sigmoidal curve for patients with HbA1c > 7% shifts to the left when compared with HbA1c  ≤  7% [5]. The electrophoresis of the patient’s sample showed the appearance of a hemoglobin F and a marked unexplained electrophoretic shift to the right into the F zone when conducting electrophoresis, as indicated in the results section.

The analytic basis for HbA1c and A0 is irrefutable, whether actual or artifactual, and the appearance of an HbF peak further complicates our findings. Nevertheless, it is known that many interfering factors of endogenous or exogenous compounds, as well physiological and biochemical conditions, can affect the protein migration patterns during capillary electrophoresis. Further, the occurrence of de novo HbF peaks, in a particular sample, can be derived endogenously or is attributable to drug-induced production exogenously or to denatured HbS or HbD species. The paucity of the literature on this effect leads us to publish our findings. HPLC, a different method than electrophoresis, has also shown a rightward shift in A1c levels [6]. HPLC can involve boronated affinity columns as the adsorbent because it specifically binds with the cis diol configuration of stable glycation on the HbA1c molecule [7]. Such adducts, commonly known as glycation adducts and crosslinks, can be identified in the blood, for example, as glycated hemoglobin A1c, A0, or in the urine [8]. The hemoglobin procedure from the accompanying instrument and reagent literature states that the mobility of the induced hemoglobin F is not different from the physiological hemoglobin F and, in the case of patients with hyperleukocytosis, the migration speed of the sample may be accelerated, causing a shift in the profile that may result in the non-recognition of the zones. Additionally, other Hb sites which can be glycated, such as to βeta-Valine-1 [9], αlphA-Lysine-61, and βeta-Lysine-66 [10], are possible additional glycation sites, which can lead to variations in the conformation of Hb molecules.

Interestingly, myeloproliferative disorders, when treated with hydroxyurea or other drugs, can interfere with migration patterns, which promote the shift in the Hb pattern from other zones, such as HbA to HbF. This result can lead to an apparent decrease in HbA1c levels. Conversely, treatment with erythropoietin and other myeloproliferative agents falsely lowers the HbA1c values. Chronic opiate use can increase HbA1c levels [11]. Further, HbF is a tumor which expressed recurrence of the fetal protein in many cancers, the so-called recapitulation of ontogeny [12].

**Table 1:** Many HbF variants and a few HbF artifacts contribute to HbF zone peaks (adapted from SEBIA inserts and operating manual [13]).

Select F Zone HbF and Other Potential Variants							
	Wilamette	Denmark	Languidic	Chiapas	Hoshida	Sardinia	Ta-Li
HbA2 Wayne	HbQ Thailand	Porto Alegre	HbG Georgia	HbG San Jose	Hb Sabine	Hb Bassett	HbA2 J-Rovigo
HbP India	Hb Burke	Hb Verdun	Hb Dunn	Hb Sassari	Hb Alabama	Hb Bunbury	Hb Les Lias
Hb Hazebrouck	Denatured Hb D-punjab	Hb Manitoba-I	Hb Manitoba-II	Hb Chapel Hill	Hb Barcelona	Hb Rampa	Hb Sawara
Hb Boyle Heights	Hb Attleboro	Hb Vanderbilt	Hb Port Phillip	Hb Hirose	Hb Tarrant	HB Abruzzo	Hb Atago
Hb Sunnybrook	Denatured HbS	Hb Deer Lodge	Hb Delficht	Hb Swan River	Hb Kansas	Hb Tak	Hb Tamano

## 2. Materials and Methods

### 2.1. SEBIA Capi 3 System A1c Determination

We utilized the SEBIA Capi 3 capillary electrophoresis system to identify and quantify glycated hemoglobin percentages. During testing, the whole blood sample (800 μL) was mixed with a hemolyzing solution in a reagent well. The hemolyzed solution was then injected into the anodic end of one of eight capillaries filled with buffer (pH 9.4). A voltage was applied, and the hemoglobin fractions then began to migrate to the cathodic end. Electrophoretic mobility is an expression of the balance of forces acting on each individual ion. The electrical force acts in favor of motion, where the frictional force acts against it. The following relationship can be observed: The higher charge and smaller size of the ion means greater mobility and lower charge, and the larger size of the ion means decreased mobility. As the hemoglobin fractions migrate, they can be directly read by a photometer at 415 nm. The hemoglobin fractions will migrate to the cathode in the following order: A2/C, E, S/D, F, A0, other hemoglobin (including some minor A1), and lastly A1c. The SEBIA Capi 3 HbA1c calculation for the measurement of HbA1c matches the following established equation by the International Federation of Clinical Chemistry.

$$\%HbA1c=100\times \left\{\frac{Area\;HbA1c}{Area\;HbA1c+Area\;HbA0}\right\}$$

### 2.2. Buffy Coat Depletion Sample Preparation

For buffy coat depletion, whole blood samples were spun down at 2000 rpm for 10 min at room temperature. Then the plasma was removed and the buffy coat was separated and removed from the sample. The plasma was placed in the capillary tube, mixed, and run on the SEBIA Capi 3 according to the manufacturer’s instructions.

### 2.3. High White Blood Cell Blood Sample Dilution and Assay Procedure

High-WBC whole blood samples were serially diluted with normal saline to assess the cut-off point of the WBC count for successfully running a sample on the SEBIA Capi 3, according to manufacturer’s instructions. Each of two samples, one with a WBC count of 370 × 10^3^/μL and the other with a WBC count of 125 × 10^3^/μL, was used in this particular set of experiments.

## 3. Results

**Figure 1:**
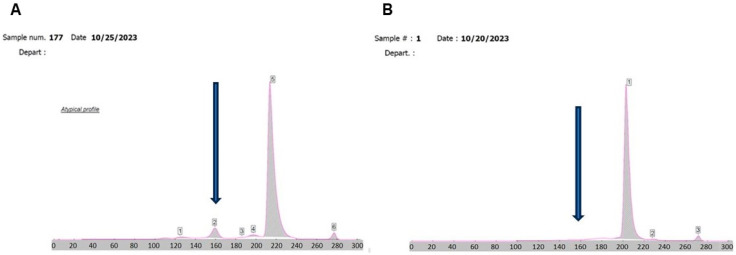
Atypical HbA1c profile of a patient with HbA1c capillary electrophoretic shift analysis using a whole blood specimen with a WBC count of 322 × 10^3^/μL. (**A**) Patient 1 sample 177 HbA1c result with noted up-field shift. (**B**) Patient 1 sample 1 HbA1c result with noted shifting zone on repeated sampling and the appearance of an HbF peak.

Four samples of the patient and one replicate on the following day (*n* = 5) were used in this study. Each patient sample had an initial HbA1c and electrophoresis result, which were obtained on the SEBIA Capi 3 instrument according to manufacturer’s instructions. The initial finding on each and subsequent sampling of HbA1c showed atypical profiles that were persistently uninterpretable and unobtainable. Capillary zone electrophoresis was uninterpretable as well due to a shift up-field, although the pattern appeared consistent (Figure 1A) when the HbA1c was run on a whole blood specimen with a WBC count of 322 × 10^3^/μL.

**Figure 2:**
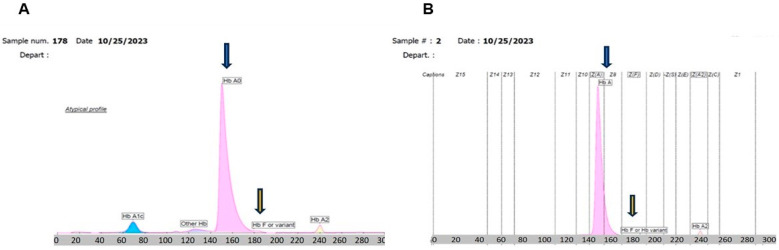
Restoration of normal HbA1c migration pattern of capillary electrophoresis by buffy coat depletion. (**A**) Patient 1 sample 178 HbA1c analysis run on whole blood specimen with a WBC count of 322 × 10^3^/μL with atypical profile after buffy coat depletion. A1c = 6.6%. (**B**) Patient 1 sample 2 capillary zone electrophoresis on whole blood specimen WBC count 322 × 10^3^/μL after buffy coat depletion showing restoration of normal zone Seven (Z7) A1c migration pattern. The migration zones are identified and within normal ranges.

**Figure 3:**
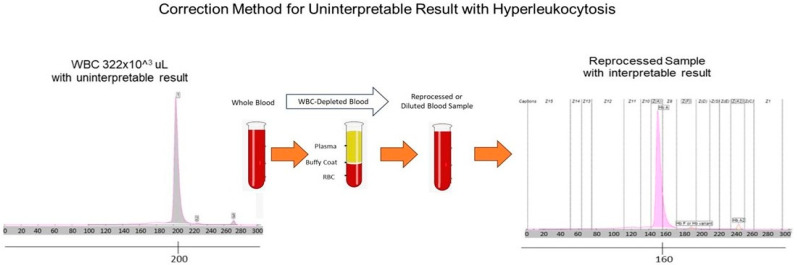
The diagram demonstrates the procedure of correction for the uninterpretable results of capillary electrophoretic shift in the HbA1c chromatograms of hyperleukocytosis patients. For buffy coat depletion, whole blood samples were spun down at 2000 rpm for 10 minutes at room temperature. And then the autologous plasma was used to resuspend the cells in the bottom. High-WBC whole blood samples were serially diluted with normal saline to assess the cut-off point of the WBC count.

Repeated and subsequent analysis of sample one and two revealed the appearance of a persistent HbF peak or variant in the normal range, which could not be verified but may be artifactual (Figure 2A). After a buffy coat depletion study was performed on this sample, patient WBC count 322 × 10^3^/μL with an atypical profile returned to a normal migration pattern for HbA1c, and the result was interpretable (Figure 2B). Capillary zone electrophoresis on the same sample after buffy coat depletion resulted in the full restoration of the HbA1c peak to the normal zone, when conducting HbA1c determination. The migration zones are identifiable and within normal ranges (Figure 3). HbA1c, HbA0, and HbA2 all normal in position and in concentration compared with the normal patient control sample. Then, reproducible electrophoresis was performed when the whole buffy coat removed via same method, as described above.

Moreover, we encountered other samples with a WBC count of 147 × 10^3^/μL, 292 × 10^3^/μL, and 116 × 10^3^/μL. Only the sample with a WBC count of 116 × 10^3^/μL could be analyzed without any further processing. However, when we encountered two more samples with WBC 370 × 10^3^/μL and 125 × 10^3^/μL, we attempted to minimally dilute the sample to ascertain where the limit of interference begins. In this set of experiments, samples were serially diluted with the addition of normal saline and then reprocessed to find where the point of interference was no longer seen (Table 2).

## 4. Discussion

We have unequivocally demonstrated that the up-field shift in A1c chromatograms was due to hyperleukocytosis interference. At the same time, it is known that HbF was not derived from the patient’s transfusions, which is not directly produced by tumor cells. What we cannot rule out is whether the HbF finding is artifactual or induced (Figure 1B). One possibility is the biological environment of cancer in nearby blood cells, or the blood cell-derived recapitulation of ontogeny mechanisms of hemoglobin origin, as in genetic mechanisms like thalassemia [14], myeloid leukemia [15], or sickle cell disease [14,16]. This de novo peak remains undetermined as to its nature or origin. Nevertheless, we suggest possible causes, including HbF inducers from pharmaceuticals (Supplementary Table S1) or natural compounds from dietary natural world food sources. These include angelicin, linear psoralens, resveratrol, and rapamycin, isolated from Streptomyces species, which can all be undocumented sources of HbF induction [17] (HbA1c = 6.6%). Other Hgb species are likely denatured Hgb. HbA1c, HbA0, and HbA2 are normal regarding the position and concentration compared with normal patients. Hgb F/variant is in the normal range, but may be an artifact [18].

While it remains unknown if the patient’s HbF induction was actual or artifactual, we may conclude that it is a putative explanation for the appearance of this peak. We know from early efforts at pharmacologic induction of HbF, centered on inducing bone marrow stress, which does involve chromatin remodeling, that HbF induction is a common phenomenon. In that regard, the induction of the HbF gene (HBG), as opposed to inhibiting gene repression enzymes for HBG, through post-translational mechanisms, also including DNA methyltransferase 1 (DNMT1), histone deacetylases (HDAC), lysine-specific demethylase 1 (KDM1A/LSD1), and protein arginine methyltransferase 5 (PRMT5), is an epigenetic measure to induce HbF production [19] (Supplementary Table S1). In terms of chromatin remodeling and epigenetic expression or repression, coactivators are opposed by corepressors, which together remodel chromatin to repress gene transcription rather than induce it. Epigenetic silencing enzymes for the HBG can include euchromatic histone-lysine methyl transferase 2 (EHMT2) and PRMT5 [19].

Notably, a second unrelated patient from our mostly aged male population was subsequently found with roughly half the white cell volume and a similar pattern in electrophoresis. A repeated depletion study was conducted, which rendered the HbA1c peak restored and interpretable. We then undertook a dilution study with normal saline and found a minimally modified sample of 125 × 10^3^/μL WBC, which was diluted with 10 μL saline to give a WBC count of 119 × 10^3^/μL, which passed without interference. However, a higher concentration of WBC, 370 × 10^3^/μL, required 70% sample dilution with saline to be interpretable. This calls into question whether dilution would suffice to make sample interpretable rather than extensive buffy coat depletion. Indeed, the sample with a WBC of 119 × 10^3^/μL had an RBC of 1.6 × 10^6^/μL and was still able to process on the SEBIA Capi 3.

## 5. Conclusions

The analytic basis for HbA1c and A0 in glycemic status is irrefutable and well-established, but endogenous factors culminating in interference or exogenous factors during treatment complicate findings, which can lead to aberrant chromatography when measured by capillary electrophoresis. This case establishes hyperleukocytosis in general as a plausible cause for aberrant A1c results. Further, the occurrence of de novo HbF peaks, in a particular sample, can be derived endogenously or is attributable to drug-induced production exogenously, or represents denatured HbS and HbD species. The paucity of literature on this effect led us to publish our findings in this case, which is a report that adds to the very limited literature published in the English language available on this topic. Moreover, it is known that many interfering species or compounds, as well as physiological and biochemical conditions, can affect the protein migration patterns during capillary electrophoresis. The issue remains how to assess glycemic control if the HbA1c result is unexpectedly low [20], inaccurate, or uninterpretable.

We suggest repeated analysis of unacceptable or confusing results, which, if they still do not process, should be followed by buffy coat depletion or minimally modifying the sample to lower the WBC with saline to counts ranging from 120 to 130 × 10^3^/µL. Our studies suggest that, with hyperleukocytosis conditions, a WBC level of 116–130 × 10^3^/µL appears to be the tolerance range under which the Capi 3 capillary electrophoresis can provide direct results. Levels higher than this would require depletion steps or dilution to achieve interpretable results. While these comments are helpful and our veteran population uniquely plentiful, the finding is typically rare and the observed literature on this topic is lacking.

## Figures and Tables

**Table 2:** Experimental findings with multiple sample dilutions to find cut-off point of WBC interference.

Initial Count	Volume of Saline Added	Resulting Count	Chromatogram
116 × 10^3^/μL	none	116 × 10^3^/μL	Normal
125 × 10^3^/μL	10 μL	119 × 10^3^/μL	Normal
370 × 10^3^/μL	100 μL	315 × 10^3^/μL	Abnormal
315 × 10^3^/μL	150 μL	250 × 10^3^/μL	Abnormal
250 × 10^3^/μL	150 μL	184 × 10^3^/μL	Abnormal
184 × 10^3^/μL	40 μL	164 × 10^3^/μL	Abnormal
164 × 10^3^/μL	30 μL	159 × 10^3^/μL	Abnormal
159 × 10^3^/μL	10 μL	155 × 10^3^/μL	Abnormal
155 × 10^3^/μL	20 μL	152 × 10^3^/μL	Abnormal
152 × 10^3^/μL	30 μL	138 × 10^3^/μL	Abnormal
138 × 10^3^/μL	10 μL	129 × 10^3^/μL	Normal

## Data Availability

All databases and software used for supporting the conclusions of the article are available upon request as are deidentified patient samples and results.

## Supplementary Materials

The following supporting information can be downloaded at https://www.mdpi.com/article/10.3390/biomedicines14010171/s1, Table S1: Select known HbF inducer drugs, small molecules by name, class, type, mechanism of action and target. References [18,21,22,23,24,25,26,27,28,29,30,31,32] are cited in the Table S1.

## Abbreviations

HbA1c	Hemoglobin A1c
HbA0	Hemoglobin A0
HbF	Hemoglobin F
HbS	Hemoglobin S
HbA	Hemoglobin A
HbD	Hemoglobin D
HbC	Hemoglobin C
CLL	Chronic Lymphocytic Leukemia
WBC	White Blood Cell
IGHV	Immunoglobulin Heavy Chain
T2DM	Type 2 Diabetes Mellitus
K+	Potassium
K-EDTA	Potassium Ethylenediaminetetraacetic Acid
EHMT2	Euchromatic Histone-lysine Methyltransferase 2
PRMT5	Protein Arginine Methyltransferase 5
KDM1A/LSD1	Lysine-specific Demethylase 1
DNMT1	DNA Methyltransferase 1
HBG	Hemoglobin F Gene
