# Supplementary material for: A Novel Aberrant HbF Peak with Electrophoretic Shift in A1c of a Patient with Chronic Lymphocytic Leukemia (CLL) Was Reversible to Give Interpretable Results
Source: Biomedicines. 2026 Jan 13;14(1):171. doi: 10.3390/biomedicines14010171 (PMC12838682; doi:10.3390/biomedicines14010171)
Supplement: Supplementary file 1 [file biomedicines-14-00171-s001.zip › biomedicines-3770057-supplementary.pdf]

Supplementary Table S1. Select known HbF inducer drugs, small molecules by name, class, type, mechanism of action and target.

HbF Inducer Drug Name (Class, Type and Target)	Drug Indication/ Molecular Mechanism	Fold Change by Western Blot	Reference
Daunorubicin Hydrochloride	Acute Myeloid Leukemia	4.55-5.76	11
Raltitrexed	Colorectal Cancer	**	11
Floxuridine	Hepatic Cancer	**	11
Epirubicin Hydrochloride	Breast Cancer	**	11
Methoxsalen	Cutaneous T-Cell Lymphoma/ Psoriasis	4.44	11
decitabine	Histone deacetylase (HDAC) inhibitors	**	12
Trichostatin-A		Epigenetic mechanism	13
GSK3482364	DNMT1 inhibitor		11
Hydroxyurea		**	11
sodium butyrate	butyrate derivatives,	**	11
Decitabine 5-azacytidine (5-aza) and 5-aza-2′-deoxycytidine Azacitidine	DNA MethylTransferase (DNMT1) inhibitors	hypomethylating agents azacitidine, decitabine and guadecitabine HDAC and DNA methyl transferase (DNMT) inhibitors	11
5-aza 2-deoxycytidine azacitidine	Histone deacetylase (HDAC) inhibitors	**	18
Trichostatin-A	Activation of P38 MAPK signaling pathway	HDAC inhibitor	18
adipicin	Activation of P38 MAPK signaling pathway	HDAC inhibitor	21
Valproic acid	Activation of P38 MAPK signaling pathway	HDAC inhibitor	18,22
Sodium 2,2 dimethyl butyrate	**	HDAC inhibitor	23,24
Scriptaid	Activation of P38 MAPK signaling pathway	HDAC inhibitor	25
Erythropoietin	Activation of STAT5, Src family kinase and ERK-1/ERK-2 MAPK signaling pathways	**	26
Fructus trichosanthis	Activation of ERK and P38 Mitogen Activated Protein signaling pathway	**	27
Suberoylanilide hydroxaminc acid	**	histone deacetylase inhibitor	27
Perphenazine	Schizophrenia	5.16	11
Fluoxetine Hydrochloride	Depression	3.25	11
Phenelzine	Depression	3.52	11
Buspar	Anxiety	3.28	11
Enalapril Maleate	Congestive Heart Failure	1.81	11
Granisetron Hydrochloride	Antimimetic	1.75	11
Thalidomide	*ß*-thalassemia /antimimetic	**	28
Digoxin	Heart Failure	3.50	11
Atenolol (beta blocker)	Hypertension	4.49	11

Benazepril Hydrochloride	Elevated Blood Pressure	3.80	11
UNC0638	euchromatic histone-lysine-*N*-methyltransferases 1 and 2 (EHMT1/2) inhibitors.	**	29,30,31
Albendazole	Anthelmintic	2.86	11
Methylprednisolone	Rheumatoid Arthritis	2.32	11
Triamcinolone Acetonide	Eczema	3.67	11
6-Azauridine 2.25 Psoriasis	Psoriasis	2.25	11
Ampiroxicam	Arthritis	4.34	
Prednisolone	Rheumatoid Arthritis / Arthritis/ inflammation/. Congenital Adrenal Hyperplasia	2.09-4.32	11
Delta1-Hydrocortisone 21-hemisuccinate Sodium	Corticosteroid-responsive Dematoses	1.90	11
Chloroxine	Seborrheic Dermatitis	2.76	11
Ebselen	Type 2 Diabetes Mellitus	1.95	11
Quinidine Hydrochloride	Cardiac Dysrhythmia	1.99	11
Pitavastatin	Elevated Cholesterol	3.92	11
Fexofenadine Hydrochloride	Seasonal Allergic Rhinitis	2.28	11
Pyrimethamine	Malarial Infection	2.23	11
Acyclovir	Viral Infection	5.14	11
Mafenide Acetate	Bacterial Infection	2.11	11
Ribavirin	Chronic Hepatitis C	2.02	11
Tetracycline	Bacterial Infection	3.95	32
Pfizerpen	Bacterial Infection	4.14	11
Cefazolin Sodium	Bacterial Infection	4.71	11
Dibenzyline	Pheochromocytoma	3.28	11
Tegaserod Maleate	Irritable Bowel Syndrome	1.93	11
